# Antidepressant-Coincident Manic Episode in a Prepubertal Girl Presenting With Obsessive-Compulsive or Related Disorders: A Case Report

**DOI:** 10.1192/bjo.2024.685

**Published:** 2024-08-01

**Authors:** Pinki Sevda, Surendra Singh Rajpurohit, Navratan Suthar, Charul Gupta, Deepika Biyyala

**Affiliations:** 1 AIIMS Jodhpur, Jodhpur, India; 2 Government Medical College, Pali, India

## Abstract

**Aims:**

Obsessive-Compulsive or Related Disorders (OCRDs) comprise a group of disorders characterized by repetitive thoughts and behaviours and are fairly less prevalent among children. The recommended treatment for OCRDs involves high doses of antidepressants, specifically selective serotonin reuptake inhibitors (SSRIs), along with non-pharmacological management. However, evidence suggests that the risk of inducing mania with antidepressants may be especially high in children and adolescents aged 14 years and younger.

**Methods:**

Here, we present a case of a nine-year girl, studying in fifth standard, with normal birth and development history, with no past/family history of psychiatric illness, presented with psychiatric illness of one-year duration and was diagnosed with Trichotillomania, Obsessive-Compulsive Disorder, Skin picking and Onychophagia as per the 11^th^ revision of International Classification of Diseases (ICD-11). After initiating tab. escitalopram 5 mg for 10 days, child developed a manic episode, which leads to a diagnostic dilemma as well as difficulties in her further management. In view of the bipolarity, escitalopram was stopped and the child was started on tab. aripiprazole 2.5 mg which was gradually up-titrated to 7.5 mg/day, following which the manic episode completely resolved and there was also improvement in OCD, hair pulling and skin picking behaviour. Later for the remaining symptoms few sessions of Habit reversal therapy were held. Currently the patient is maintaining well on aripiprazole 7.5 mg for the last six months.

**Results:**

The uniqueness of this case is demonstrated through current limited literature on comorbid OCRDs and antidepressant coincident manic episode, especially in children in whom diagnosing manic episode possess a great challenge owing to various differential diagnosis. While deciding pharmacological therapy in children with OCRDs or Mania the efficacy as well as their safety profile should be considered. Currently there are no medications approved by FDA for treatment of acute manic episode in patients below 10 years of age and use of SSRI which are considered first line for treatment of some OCRDs may exaggerate the manic episode. In literature, second generation antipsychotics such as aripiprazole is found to be useful for the management of both manic episode (as monotherapy) as well as OCD (as an adjuvant). In this case aripiprazole monotherapy led to significant improvement in both groups of symptoms.

**Conclusion:**

Thus, SSRIs should be used cautiously in children with OCRDs and aripiprazole along with other approved non-pharmacological management strategies can be considered as a good treatment option in children with OCRDs and anti-depressant coincident manic episode.

